# Metabolomics Applications in Children: A Right Way to Go

**DOI:** 10.3390/metabo10090364

**Published:** 2020-09-08

**Authors:** Maria Elisabetta Baldassarre, Nicola Laforgia

**Affiliations:** Department of Biomedical Science and Human Oncology-Neonatology and NICU Section, University “Aldo Moro” of Bari, 70124 Bari, Italy; nicola.laforgia@uniba.it

**Keywords:** metabolomics, newborns, hypoxic–ischemic encephalopathy, intrauterine growth restriction, congenital infections, genetic diseases, neonatal nutrition

## Abstract

Metabolomics is a new science based on the study of the metabolome, representing the set of all the metabolites of a biological organism, which are the final products of its gene expression. Metabolomics appears to be a promising tool in perinatal studies, such as hypoxic–ischemic encephalopathy (HIE), intrauterine growth restriction (IUGR), congenital infections, genetic diseases, neonatal nutrition.

## 1. Introduction

Metabolomics is a relatively new science based on the systematic study of the chemical footprints left by a specific cellular process. The metabolome represents the set of all the metabolites of a biological organism, which are the final products of its gene expression. While the data of mRNA-gene expression and proteomic analyses do not fully explain what could happen in a cell, the metabolic profile can provide a “snapshot” of that cell’s physiology. The metabolic profile in human biological fluid is the result of the interaction between the human genome and the micro/macro environment. The most important microenvironment for humans is the microbiota. Metabolomics has the extraordinary potential of exploring the whole collection of metabolites produced also from the commensal communities of microorganisms. Worldwide research projects are attempting to map the human microbiome, giving insight into new species and genes and revealing new roles of the microbiome in human health and disease. The evidence of the interplay between intestinal commensal bacteria and host physiological functions has grown over the last years, shedding new light on clinical research on pathological conditions [1]. One of the challenges of systemic biology is to integrate proteomics, transcriptomics and metabolomics information to have a more complete overview of living organisms. Metabolomics appears to be a promising tool in perinatal studies, such as hypoxic–ischemic encephalopathy (HIE), intrauterine growth restriction (IUGR), congenital infections, genetic diseases, neonatal nutrition. 

## 2. Hypoxic–Ischemic Encephalopathy (HIE)

HIE is among the most relevant contributors to neurological disability in the pediatric age. HIE has been associated with damage to the integrity of the blood–brain barrier and, hence, brain injury could be reflected in peripheral blood samples. To date, the lack of early, reliable and specific biomarkers has hampered a more effective use of therapeutic hypothermia (TH), started within 6 h of birth, as the standard of care for infants with moderate or severe HIE, reducing mortality and improving neurodevelopmental outcome in survivors. Comprehensive metabolic phenotyping has been increasingly employed for identifying possible therapeutic targets, and the discovery of biomarkers to support treatment selection, patient stratification and enhanced diagnosis. The need to know the biological modifications underlying perinatal asphyxia and hypoxic–ischemic encephalopathy needs new diagnostic and therapeutic tools. Metabolomics according to its own features is a powerful approach that may help in the identification of specific metabolic profiles related to the pathological mechanism and foreseeable outcome. An extensive review [2] of the relevant literature provides data regarding the metabolomic profiles of animal and human infants exposed to perinatal asphyxia or developing hypoxic–ischemic encephalopathy, leading to the identification of promising metabolomic markers. Most of the studies in human newborns focused on the urinary metabolome. The author’s conclusions are that “although the chase of a set of biomarkers or of a metabolomics phenotype seems to be promising, the goal is still ‘elusive’”.

A new study [3] evaluating early evolving blood metabolome provides, for the first time, useful data to identify newborns who will show pathologic Magnetic Resonance Imaging (MRI) patterns after TH. The authors demonstrated changes of purines (i.e., xanthine and xanthosine), as well as of the steroid hormone biosynthesis pathway, in newborns with pathologic MRI outcomes in blood samples drawn before the initiation of TH during the whole study period. The authors suggested the development of adjuvant therapies to be combined with TH, i.e., allopurinol and steroid hormones.

## 3. Intrauterine Growth Restriction (IUGR)

IUGR refers to a condition in which a fetus is unable to achieve its genetically determined potential size. IUGR newborns are exposed to short- and long-term health problems (i.e., cardiometabolic syndrome). Biomarkers capable of identifying this condition early on, and stratifying its severity both pre- and postnatally, are still lacking.

An interesting review [4] showed that metabolomic studies regarding the IUGR condition clearly differentiate between IUGR babies and controls and, despite all limitations, suggest an “early” pattern of glucose intolerance, insulin resistance, catabolite accumulation, disrupted amino acid metabolism and abnormal fetal liver function.

Metabolomics could also help neonatologists to improve the quality and appropriateness of IUGR newborns’ growth, detecting nutritional deficiencies of several macro- (proteins, lipids) and micronutrients (calcium, iron, phosphate). 

## 4. Congenital Infections

The most common congenital infection in the developed countries is human Cytomegalovirus infection (cHCMV), associated with significant long-term neurological or audiological impairment. Antiviral treatment improves the audiological and neurodevelopmental outcomes in symptomatic patients. The diagnosis of asymptomatic cases at birth and the identification of those who will develop long-term sequelae is an area of strong clinical need, because it would ensure that these babies receive prompt treatment and follow-up, to improve their clinical prognosis. A very recent “in vivo” study [5] established a metabolic fingerprint associated with HCMV infection that included alanine, betaine, dimethylamine and glycine, all reduced in cHCMV infected newborns compared to controls. Dimethylamine and glycine can originate from the metabolic degradation of betaine, associated to one-carbon metabolism as a source of one-carbon unit. The authors would suggest that a reduction in one-carbon metabolism in HCMV infected infants could explain their cognitive impairment.

## 5. Genetic Diseases

Phenylketonuria (PKU) is a rare autosomal recessive condition affecting about 1 in 10,000 people in Europe, with a higher rate in some countries, such as Ireland and Italy. Newborns affected by PKU are generally identified through newborn screening (NBS) programs and expanded NBS based on tandem mass spectrometry (MS/MS), which allows the diagnosis through the quantification of Phe and the Phe/Tyr ratio in dried blood spot (DBS) samples.

Prematurity, birth weight, blood transfusion and total parental nutrition (TPN) can influence NBS results. TPN frequently causes false positives for amino acids at NBS in MS/MS, and follow-up is strongly recommended, until the end of the TPN procedure. A recent case report [6] showed that Phe/Tyr ratioby MS/MS could be useful during the administration of parental nutrition rich in AAs in the neonatal period for the diagnosis of Hyperphenylalaninemia (HPA), a known variant of PKU diagnostic suspicion. This case shows that many biomarkers may better describe a metabolic disease than a single marker, perfectly fitting with the “omics” and the “personalized medicine” era. This era presents a real perspective to further expand NBS to many other metabolites and disorders, improving the metabolic fingerprint description in newborns. 

Congenital adrenal hyperplasia (CAH) is characterized by defects in steroid biosynthesis, mostly due to a 21-hydroxylase deficiency (21-OHD) as a consequence of CYP21 gene mutations [7]. A neonatal case report [8] demonstrated that the application of a steroid profile by liquid chromatography–tandem mass spectrometry (LC–MS/MS) quickly revealed the related 21-OHD steroid alteration, allowing for a hormone replacement therapy in the first days of life.

## 6. Nutrition 

The application of metabolomics can provide new insights into the field of infant nutrition. 

### 6.1. Human Milk

Human milk (HM) components influence growth, maturation of the immune system, protection from toxins and pathogens, cognitive development and the establishment of the intestinal microbiota. NMR-based metabolic profiling can provide a rapid evaluation of breast milk composition with better understanding of its nutritional properties. The results of the first metabolomic study on HM, using both NMR spectroscopy and gas chromatography-mass spectrometry (GC-MS), were published in 2012 [9] and further evaluated with NMR spectroscopy by Praticò et al. [10]. 

A new review [11] analyzed several maternal and infant-related factors and the different composition of human milk metabolome (HMM), which relates to different physiological conditions such as maternal weight, maternal diet and education, geographical location, ethnicity, type of delivery, gestational age, stage and course of lactation, as well as to some diseases such as mastitis, human immunodeficiency virus (HIV) infection and chemotherapy.

### 6.2. Metabolic Diseases

Glycogen storage diseases (GSD) are hereditary metabolic disorders, classified from GSD type I to GSD type XI, caused by deficiency of one of the enzymes involved in glycogen metabolism. A recent study [12] investigated the impact of the life-long cornstarch-rich diet in GSD-1 patients. The clinical onset of GSD-I usually occurs in the first year of life, during complementary feeding, with symptoms related to severe fasting hypoglycemia, hepatomegaly, failure to thrive and growth retardation.

The authors evaluated if this peculiar diet could impact substrate availability for microbial fermentation, affecting the production of short chain fatty acids (SCFAs). Their results indicate that GSD-1 patients have increased pro-inflammatory bacteria and reduced beneficial species and that a specific diet does not seem to help the composition of gut microbiota, as the anti-inflammatory bacteria are not sufficient to counterbalance the dysbiosis.
